# Association of the single nucleotide polymorphism in chromosome 9p21 and chromosome 9q33 with coronary artery disease in Chinese population

**DOI:** 10.1186/s12872-017-0685-0

**Published:** 2017-09-30

**Authors:** Qi Li, Wenhui Peng, Hailing Li, Jianhui Zhuang, Xuesheng Luo, Yawei Xu

**Affiliations:** 1 No. 113 Hospital of Chinese People’s Liberation Army (PLA), Ningbo, China; 2 Shanghai Tenth People’s Hospital, Tongji University School of Medicine, Shanghai, China

**Keywords:** Coronary artery disease, Single-nucleotide polymorphisms, rs1333049, rs702548

## Abstract

**Background:**

Our study aims to explore the association of rs7025486 single-nucleotide polymorphisms (SNP) in DAB2IP and rs1333049 on chromosome 9p21.3 with the coronary artery disease in Chinese population.

**Methods:**

All patients came from the east China area and underwent coronary angiography. Rs7025486 and rs1333049 polymorphism were genotyped in 555 patients with CAD and in 480 healthy controls that underwent coronary angiography.

**Results:**

In Chinese population, the rs7025486 genotype in the case group was no significant different than the control group (*P* = 0.531).Meanwhile, the rs1333049 SNP has statistically significant (*P* = 0.006), which was the independent risk factors for CAD (OR1.252, *P* = 0.039), and consistent with the past studies conclusion.

**Conclusion:**

Genotype of rs1333049 on chromosome 9p21, but not rs7025486 on chromosome 9q33, is an independent determinant of the incidence of CAD in Chinese population.

## Background

Coronary artery disease (CAD) is a chronic multifactor inflammatory disease which can progress to acute coronary syndrome and sudden cardiac death [1]. CAD is associated with a family history as well as several established risk factors including diabetes mellitus, hypertension, hyperlipidemia and smoking, suggesting that the pathogenesis of CAD has a substantial genetic component [2]. Genome-wide association studies (GWAS) have identified several genetic variants that increased susceptibility to CAD and acute myocardial infarction (AMI) in the primary prevention setting [3]. The strongest association signal in the genome in GWAS studies for CAD and AMI that has been published thus far comes from a number of single-nucleotide polymorphisms (SNPs) with a high degree of linkage disequilibrium between each individual on chromosome 9p21 [4–6]. However, several subsequent studies have shown inconsistent results when the association between these genetic variants and CAD or AMI was examined [7, 8]. In addition, these data are mostly from American African, Caucasian, South Korean, and Japanese cohorts. Most of SNPs on 9p21 are located within a long non-coding RNA, namely antisense non-coding RNA in the INK4 locus (ANRIL). It seems plausible that the influence of ANRIL on CAD is mediated by the upstream genes CDKN2A and CDKN2B. The dysfunction of CDKN2A and CDKN2B subsequently causes excessive cell proliferation [9–11].

Recently, a European GWAS reported that chromosome 9q33 contains a novel susceptibility locus DAB2IP associated with abdominal aortic aneurysm, early onset myocardial infarction, peripheral artery disease and pulmonary embolism [12]. Nevertheless, relevant reports on the association of rs7025486 located within 9q33 with CAD were lacking in Chinese population. DAB2IP is considered as a Ras-GTPase activator and a tumor suppressor gene, repressing tumor proliferation and metastasis and maintaining chromosomal stability [13, 14].

Toward this end, we selected two SNPs, which represented the most associated and independent SNP rs1333049 at 9p21.3 and rs7025486 at 9q33 in the previous studies, to investigate their role in predicting CAD.

## Methods

### Study population

The study protocol was approved by the hospital ethics committee, and written informed consents were obtained from all subjects. The study population consisted of 1151 Chinese Han patients undergoing coronary angiography to evaluate suspected or established CAD. Sixty-four patients with type 1 diabetes mellitus were identified by measuring C peptide levels and excluded; we also excluded 52 patients with chronic viral or bacterial infections, tumors, or immune system disorders. Of the final 1035 enrollments, 555 patients had significant CAD (≥50% luminal diameter narrowing in at least one coronary artery), and 480 were considered to be healthy controls [15]. Type 2 diabetes mellitus was referred to as a fasting plasma glucose level of ≥7.0 mmol/L or a non-fasting plasma glucose level of ≥11.1 mmol/L, or taking oral hypoglycemic drugs or receiving parenteral insulin therapy [15]. Patients were diagnosed with hyperlipidemia if they had serum levels of total cholesterol (TC) >5.7 mmol/L (220 mg/dl), low-density lipoprotein cholesterol (LDL-C) >3.64 mmol/L (140 mg/dl), triglycerides (TG) >1.7 mmol/L (150 mg/dl) or high-density lipoprotein cholesterol (HDL-C) <0.91 mmol/L (35 mg/dl). Early-onset CAD was considered as clinical CAD occurring by age ≤ 55 years in male or ≤60 years in female patients [9].

### Genotype determination

Genomic DNA was extracted from peripheral blood cells according to the manufacturer’s instrument (Tiagen Biotech, China) [15]. Genotyping was performed with TaqMan SNP allelic discrimination by means of an ABI 7900HT (Applied Biosystems, Foster City, CA, USA), in a 384-well format [16]. The TaqMan Assay kit was obtained from Applied Biosystems (Foster City, USA). Genotypes were determined as previously described [16]. Finally, we applied the ABI Prism SDS software version 2.1 to analyze the data.

### Statistical analysis

Continuous variables are expressed as mean ± standard deviation, while categorical data are presented as frequencies or percentages. The differences among groups were determined by Student t test or one-way ANOVA analysis [16]. The differences in categorical data among groups were determined by the chi-square test. Odds ratios (*ORs*) of CAD for CC genotype on rs1333049, and other risk factors, were estimated by multivariate Logistic regression analyses [17]. A 2-sided probability level of ≤0.05 was considered significant. All analyses were performed with SPSS for Windows 13.0 (SPSS Inc., Chicago, Illinois, USA).

## Results

### Baseline characteristics of enrollments

The baseline characteristics of the study groups are presented in Table 1, which show the expected differences in classical risk factors in CAD patients compared with controls. CAD patients in our studies are predominance of male subjects (62.52%). Furthermore, patients with CAD have higher incidence rate of smoking and diabetes mellitus compared with healthy controls (43.96% vs. 20.42% and 67.39% vs. 31.25%, respectively). There are no differences in age, history of hypertension and hyperlipidemia, family history of CAD, and levels of blood urine nitrogen (BUN) and uric acid between two groups.

**Table 1:** Baseline clinical characteristics and biochemical assessments

	CAD (*n* = 555)	Control (*n* = 480)	*p*-value
Male	62.52	50.21	*<0.001*
Age, yrs	62.94 ± 9.68	61.97 ± 9.83	0.11
Smoking	43.96	20.42	*<0.001*
BMI, kg/m^2^	24.37 ± 3.14	25.63 ± 3.14	*0.034*
Hypertension	78.37	78.65	0.923
Diabetes mellitus	67.39	31.25	*<0.001*
Hyperlipidemia	18	13.7	0.101
Family history	6.3	3.8	0.063
SBP, mmHg	133 ± 20	134 ± 19	0.387
DBP, mmHg	79 ± 12	81 ± 11	*0.008*
Total cholesterol, mmol/L	4.53 ± 1.10	4.67 ± 0.99	*0.031*
Triglyceride, mmol/L	1.93 ± 1.48	1.79 ± 1.23	0.096
HDL, mmol/L	1.11 ± 0.29	1.26 ± 0.30	*<0.001*
LDL-C, mmol/L	2.68 ± 0.92	2.69 ± 0.76	0.943
ApoA, g/L	1.20 ± 0.19	1.28 ± 0.20	*<0.001*
ApoB, g/L	0.91 ± 0.25	0.88 ± 0.22	0.054
LPA, g/L	0.28 ± 0.49	0.24 ± 0.49	0.286
Fasting glucose, mmol/L	6.81 ± 2.49	5.60 ± 1.66	*<0.001*
Creatinine, mg/L	88.94 ± 35.15	80.04 ± 22.53	*<0.001*
BUN, mmol/L	5.95 ± 2.87	5.58 ± 1.78	*0.019*
Uric acid, umol/L	318.90 ± 88.95	314.39 ± 79.32	0.415
HWE (rs7025486)	*P* = 0.840	*P* = 0.861	
HWE (rs1333049)	*P* = 0.237	*P* = 0.306	

Values are mean ± SD or n (%)

*BMI* Body mass index, *BUN* Blood urine nitrogen, *CAD* Coronary artery disease, *HDL* High-density lipoprotein cholesterol, *LDL-C* Low-density lipoprotein cholesterol, *LPA* Lipoprotein A, *HWE* Hardy-Weinberg equilibrium

*P*-values of risk factors with significance are presented as italic form

### Distribution of rs1333049 and rs7025486 genotype between CAD patients and controls

The observed rs1333049 and rs7025486 genotype frequencies did not deviate from Hardy-Weinberg equilibrium (Table 1, *P* > 0.05 using a chi-squared goodness-of-fit model). This indicated that the case and control groups were representative of the population and had no selection bias. Table 2 shows the distribution of rs1333049 and rs7025486 genotype in our study. The distribution of rs1333049 genotypes significantly differed between CAD patients and healthy controls (*P* = 0.006). In contrast, no discrepancy was found in the distribution of rs7025486 genotype between two groups (*P* = 0.531). The whole enrollments were then divided into several subgroups according to the presence of history of diabetes mellitus, hypertension, AMI and early onset myocardial infarction. However, we could not find a positive association of rs7025486 genotype with these conditions (Table 3).

**Table 2:** Genotyping of rs7025486 and rs1333049

Genotype	rs7025486 (n)			rs1333049 (n)		
	AA	AG	GG	GG	CG	CC
CAD (n = 555)	47	254	254	118	239	198
Control (n = 480)	44	203	233	128	223	129
p-value	0.531			0.006		

Data are presented as the number of patients with indicated genotype in each cell. We use the chi-square test to investigate the genotype distributions between the CAD and control group for significant deviation from those found in samples in Hardy-Weinberg equilibrium

*CAD* coronary artery disease

**Table 3:** Association between rs7025486 genotype and diabetes mellitus, hypertension, AMI and Early-onset MI

Genotype	rs7025486(n)			*p*-value
	AA	AG	GG	
Diabetes mellitus				
Yes	40	241	243	0.281
No	51	216	244	
Hypertension				
Yes	52	296	320	0.245
No	17	91	75	
AMI				
Yes	29	137	134	0.578
No	62	320	353	
Early-onset MI				
Yes	11	51	43	0.474
No	36	203	211	

Data are presented as the number of patients with indicated genotype in each cell. We use the chi-square test to investigate the genotype distributions in diabetes mellitus, hypertension, AMI and Early-onset MI groups for significant deviation from those found in samples in Hardy-Weinberg equilibrium

AMI acute myocardial infacrtion

### Rs1333049 is an independent determinant of the incidence of CAD

To gain further insight into the role of rs1333049 in independently predicting CAD, multivariate logistic regression was performed to further analyze the data by adjusting for gender, age, history of smoking, diabetes mellitus, fasting glucose, hypertension and hyperlipidemia. As shown in Table 4, apart from established risk factors including smoking, diabetes mellitus and hypertension, rs1333049 remained associated with the incidence of CAD, suggesting that rs1333049 genotype was an independent determinant of the incidence of CAD.

**Table 4:** Multivariable analysis of independent determinants for CAD

	OR	95%CI	p-value
Male	0.93	0.645–1.340	0.697
Age	1.006	0.989–1.023	0.471
Smoking	3.238	2.144–4.890	<0.001
rs1333049	1.252	1.011–1.550	0.039
Diabetes mellitus	4.26	2.952–6.149	<0.001
Hypertension	1.633	1.104–2.413	0.014
Hyperlipidemia	1.073	0.703–1.637	0.745
Fasting glucose	1.168	1.168–1.276	0.001

Gender, age, history of smoking, rs1333049, diabetes mellitus, fasting glucose, hypertension and hyperlipidemia enter into multivariate analysis

*CI* Confidence interval, *OR* odd ratio

## Discussion

Since the fact that CAD and other vascular diseases share common risk factors, including genetic variants and environmental risk factors associated with peripheral vascular disease are expected candidates affecting the risk of CAD [18, 19]. In our case–control study, two previously reported SNPs representing the genetic variants on 9p21 and 9q33 were chosen to investigate the association with CAD. Our study validated that rs1333049 at chromosome 9p21 showed a significant association with CAD, whereas variant at 9q33 showed no association with CAD and main cardiovascular risk factors in our data.

Accumulating evidence came to conclusion that the incidence of hypertension and diabetes mellitus, higher inflammatory response and LDL levels are the risk factors for atherosclerotic progression [20]. Furthermore, based on optical coherence tomography, cholesterol, hs-CRP and pentraxin 3 were associated with thin-cap fibroatherma, which is known as vulnerable plaques [20–22]. Similarly, some of these known risk factors were referred to as independent determinants of CAD in our study.

In the past decades, a number of novel susceptibility genes of CAD were identified using GWASs [19]. In particular, several SNPs on chromosome 9p21 identified by the Welcome Trust Case Control Cohort study (WTCCC), McPherson et al. [4] and Helgadottir et al. [3] met the criteria for genome-wide association. Consistent with these results, SNP rs1333049, which has been reported in other Asian population, is strongly associated with CAD in our case-control study and could be regarded confirmatory [23–25]. Based on multivariate analysis, we further collaborated that rs1333049 genotype is not secondary drift to other cardiovascular risk factors and an independent determinant of the incidence of CAD.

A mechanism behind the link between risk alleles on chromosome 9p21 and cardiovascular disease is actively being explored. Recent studies have reported that the expression of the upstream genes *CDKN2A* and *CDKN2B* as well as the long non-coding *ANRIL* was considered to be linked with the risk genotype [9, 10]. None of the SNPs in the haplotype block harbor in the transcribed regions, and thus a change of expression level ascribed to alteration of a promoter or enhancer region is indeed a plausible hypothesis. On the other hand, targeted depletion of the non-coding interval on human chromosome 9p21 in mice provided direct evidence that the risk interval has an important role in regulation of cardiac CDKN2A and CDKN2B expression, suggesting that the region modulates CAD progression by altering the dynamics of vascular cell proliferation [11].

On the other hand, no association between variant in rs7025486 and the incidence of CAD was observed, while the GWAS and replicating study consistently found the effects of a common variant in *DAB2IP* (rs7025486) on the development of CAD and other complications [12, 26, 27]. In cancer cells, DAB2IP, as a Ras-GTPase, exerted a suppressive effect on tumor invasion and maintained chromosomal stability [13, 14, 28]. However, the molecular mechanisms of DAB2IP in regulating the progression of atherosclerosis need further investigation. Leading explanations for these discrepant findings include the presence of different ethnic groups as well as relative small sample size. In fact, the frequencies of the risk-association alleles in chromosome 9q33 are similar in American African and Caucasian populations, but substantially lower in Asian descent. Thus, failing to identify any significant association of rs7025486 with the incidence of CAD in Asian populations could be attributed to substantially lower statistical power caused by the relatively lower prevalence of the risk allele. In addition, study design or small sample size may also affect the results. This is a hospital-based study in nature. In this regard, the participants in control group may not absolutely healthy and the overestimation of the proportion of healthy participants could result in selection bias in our study.

## Conclusions

Genotype of rs1333049 on chromosome 9p21, but not rs7025486 on chromosome 9q33, is an independent determinant of the incidence of CAD in Chinese population.

## Abbreviations

AMI: Acute myocardial infarction
ANRIL: Antisense non-coding RNA in the INK4 locus
CAD: Coronary artery disease
CDKN2A/2B: Cyclin dependent kinase inhibitor 2A/2B
DAB2IP: DAB2 interacting protein
GWAS: Genome-wide association studies
ORs: Odds ratios
SNPs: Single-nucleotide polymorphisms
WTCCC: Welcome trust case control cohort study
